# Associations between hyperhomocysteinemia and the presence and severity of acute coronary syndrome in young adults ≤ 35 years of age

**DOI:** 10.1186/s12872-021-01869-y

**Published:** 2021-01-23

**Authors:** Jiayin Sun, Wei Han, Sijing Wu, Shuo Jia, Zhenxian Yan, Yonghe Guo, Yingxin Zhao, Yujie Zhou, Wei Liu

**Affiliations:** Department of Cardiology, VIP Ward, Beijing Anzhen Hospital, Capital Medical University, Beijing Institute of Heart Lung and Blood Vessel Disease, Beijing Key Laboratory of Precision Medicine of Coronary Atherosclerotic Disease, Clinical Center for Coronary Heart Disease, Capital Medical University, Beijing, 100029 China

**Keywords:** Acute coronary syndrome, Hyperhomocysteinemia, Severity, Young

## Abstract

**Background:**

The prevalence of acute coronary syndrome (ACS) continues to increase among young Chinese adults. Homocysteine (HCY) has been suggested as a promoter of atherosclerosis leading to coronary artery disease (CAD). Yet, it remains uncertain whether HCY is associated with the ACS and the severity of coronary artery stenosis in young adults.

**Methods:**

Young patients (18–35 years of age) diagnosed with ACS who underwent coronary angiography (CAG) at Anzhen Hospital between January 2013 and June 2019 were assigned to the ACS group. As confirmed by CAG during the same period, an equivalent age-matched population without CAD was assigned to the non-CAD group. A serum HCY level > 15 µmol/L was defined as hyperhomocysteinemia (HHCY). The Gensini score assessed the severity of coronary artery stenosis.

**Results:**

A total of 1103 participants, including 828 ACS patients and 275 non-CAD subjects, were enrolled in this study. Young ACS patients had higher level of serum HCY and greater prevalence of HHCY compared with non-CAD subjects [for HCY, 16.55 (11.93–29.68) vs 12.50 (9.71–17.42), *P* < 0.001; for HHCY prevalence, 62.08% vs 26.18%, *P* < 0.001]. Multivariate logistic regression analysis with the stepwise method indicated that HHCY was an independent predictor associated with the presence of ACS, after adjusting for traditional confounders (OR, 4.561; 95% CI, 3.288–6.327; *P* < 0.001). Moreover, young ACS patients with HHCY had increased prevalence of ST-segment elevation myocardial infarction (STEMI) (*P* = 0.041), multi-vessel disease (*P* = 0.036), and decreased value of left ventricular ejection fraction (LVEF) (*P* = 0.01). Also, the HCY level was significantly correlated with Gensini Score in ACS patients (r = 0.142, *P* < 0.001).

**Conclusion:**

HHCY is significantly associated with the presence of ACS and the severity of coronary artery stenosis in young adults ≤ 35 years of age.

## Introduction

Acute coronary syndrome (ACS) has become a significant public health problem and the leading cause of morbidity and mortality in the entire world as well in China [1]. Although ACS primarily occurs in older people, the incidence of ACS has been gradually increasing among younger Chinese individuals aged ≤ 45 years [2]. Several traditional risk factors for coronary artery disease (CAD), which include current smoking status, elevated body mass index (BMI), and a family history of premature acute myocardial infarction (AMI), have been associated with younger age [3]. In addition, non-traditional risk factors, such as hyperhomocysteinemia (HHCY), have also been suggested as novel markers for CAD and are supposed to be added to Framingham Risk Factors (FRFs) to boost their predictive value [4, 5]. In an observational study conducted in elderly patients undergoing coronary angiography (CAG), the elevation of homocysteine (HCY) level was closely associated with severity of coronary artery stenosis [6]; still, the impact of HHCY on ACS in young adults has not drawn much attention among research community due to the relatively low prevalence of ACS among young adults.

Since general HHCY prevalence has increased over the last two decades in China [7], as well as prevalence among young individuals, the aim of the current study was to analyze the association between HHCY and ACS, including the presence and the severity of coronary artery stenosis among young adults who are 35 years of age and younger.

## Methods

### Study population

In this single-center observational study, young patients (18–35 years of age) diagnosed with ACS who underwent coronary angiography (CAG) at Anzhen Hospital between January 2013 and June 2019 were assigned to the ACS group. An equivalent age-matched population who underwent CAG for suspected CAD during the same time period at the center, but were finally confirmed as not having the coronary disease, were assigned to the non-CAD group. Participants who met any of the following exclusion criteria were excluded from the study: (1) missing homocysteine data; (2) repeated hospitalization; (3) moderate renal impairment (an estimated glomerular filtration rate [eGFR] < 60 mL/min per 1.73 m^2^), pernicious anaemia, hypothyroidism, various cancers, psoriasis; (4) myocarditis, cardiomyopathy, valvular heart disease, congenital heart disease, infective endocarditis, multiple arteritis, Kawasaki disease, rheumatic heart disease; (5) vitamin or folate supplementation within 3 months.

This study was approved by the Institutional Ethics Committee at Beijing Anzhen Hospital. The data used in the study were retrospectively obtained from electronic medical records.

### Data collection and related definitions

Baseline fasting venous blood samples were collected from all participants and the level of HCY and other laboratory indicators, such as triglycerides (TG), total cholesterol (TC), low-density lipoprotein cholesterol (LDL-C), high-density lipoprotein cholesterol (HDL-C), uric acid (UA), creatinine and high-sensitivity C-reactive protein (hs-CRP) were analyzed. HCY was measured by a Beckman Coulter AU5400 automatic biochemical analyzer using an HCY commercial kit (enzymatic cycling method). LDL-C was tested through direct LDL-C assays. eGFR was calculated with the MDRD formula according to the age, creatinine and gender of the patients. According to the testing results, TG ≥ 1.7 mmol/L was considered as hypertriglyceridemia, TC ≥ 5.2 mmol/L was considered as hypercholesterolemia, LDL-C ≥ 3.4 mmol/L was considered as a high LDL-C level, and HDL-C < 1.0 mmol/L was considered as a low HDL-C level [8]. In addition, HHCY was defined as HCY level > 15 μmol/L [9], while hyperuricemia was defined as UA level ≥ 420 mmol/L in males and ≥ 357 mmol/L in females [10].

Participants’ demographic and clinical data were collected from electronic medical records. Hypertension was defined as a systolic pressure (SBP) ≥ 140 mmHg and/or diastolic pressure ≥ 90 mmHg, or currently taking antihypertensive medications [11]. DM was defined as FBG ≥ 7.0 mmol/L and/or random glucose level ≥ 11.1 mmol/L or previously diagnosed DM treated with diet, oral agents, or insulin [12]. Familial hypercholesterolemia (FH) was defined by satisfying two or more of the following criteria: LDL-C ≥ 4.68 mmol/L, tendon/skin xanthomas, and FH history or family history of premature CAD [13]. Smoking status was defined as occasional or regular smoking ≥ 1cig/day, and former smokers with a cessation period ≤ 1 year were also included [14]. Drinker was defined as someone with an average alcohol intake ≥ 50 g/day.

All participants received coronary angiography via standard techniques. Major coronary vessels, including the left main, left anterior descending, left circumflex, right coronary artery, and main branches with a more than 2.0 mm diameter, were evaluated. Major coronary arteries with luminal diameter stenosis ≥ 50% were considered as a lesion coronary artery. Left main stenosis ≥ 50% was considered as a double-vessel disease. Moreover, ACS diagnosis was determined by the European Society of Cardiology 2015 guidelines [15]. Young ACS patients were divided into AMI and unstable angina pectoris (UAP) groups according to the clinical diagnosis, single-vessel and multi-vessel groups according to the number of lesion vessels, left ventricular ejection fraction (LVEF) ≥ 50% and LVEF < 50% groups according to the cardiac function. The severity of coronary artery stenosis was evaluated by the Gensini Score [16].

### Statistical analysis

Statistical software SPSS 22.0 (IBM-SPSS Inc., Chicago, USA) was used to conduct all the analysis. The normality of data was evaluated by the Kolmogorov–Smirnov test. Accordingly, continuous variables with normal distribution were expressed as mean ± standard deviation (SD) and compared between two groups using the independent samples *t*-test. Otherwise, data were expressed as the median and interquartile range (IQR) in case of skewed distribution, and the Mann–Whitney U test determined differences between the two groups. Categorical variables were presented as counts and percentages (%) and compared using the Chi-square test. The relationship between serum HCY level and Gensini Score was evaluated using Spearman analysis. Univariate logistic regression analysis was performed first, then variables with a *P*-value < 0.2 were selected and added into multivariate logistic regression model using the stepwise method (entry, 0.05; removal, 0.05) so as to determine their independent risk associated with ACS, which was calculated by odds ratio (OR) with 95% confidence intervals (95% CI). A value of *P* < 0.05 in a two-sided test was considered statistically significant. A power test was conducted by Power/Sample Size Calculator online.

## Results

### Baseline clinical characteristics

**Fig. 1:**
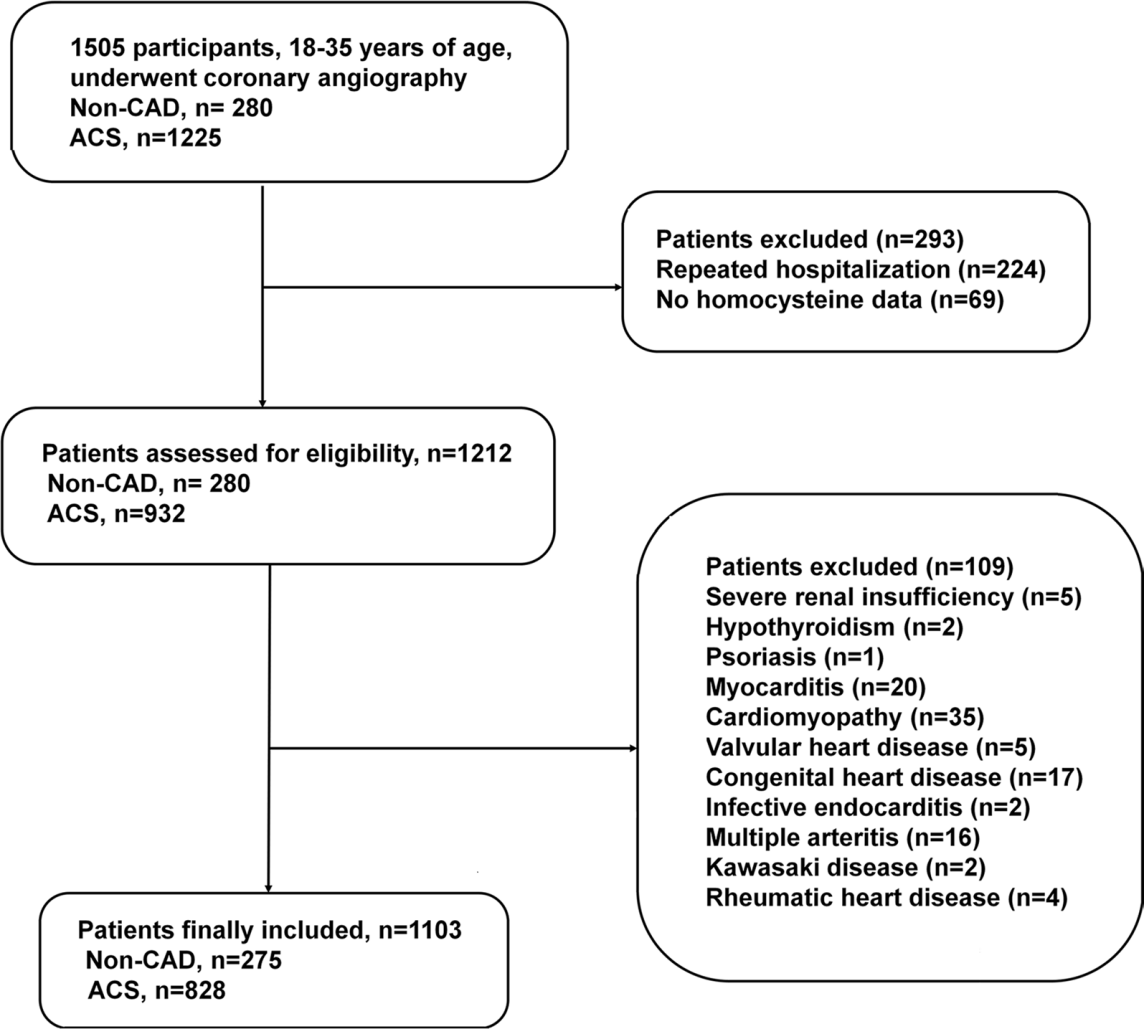
Flow chart illustrating the process of a participant enrolled in the study

A total of 1103 participants, including 828 ACS patients and 275 non-CAD individuals, were enrolled in this study. The flowchart of the study is shown in Fig. 1. Clinical characteristics and biochemical findings of involved participants are listed in Table 1. The majority of young patients with ACS were male (96.01% vs. 89.09%, *P* < 0.001). The higher prevalence of current smoker status, hypertension, DM, family history of CAD, and familial hypercholesterolemia was found in the ACS group compared to the non-CAD group. ACS patients also had higher HR, BMI, and increased levels of FBG, HbA1c, TG, TC, LDL-C, UA as well as hs CRP. Moreover, there was a greater percentage of patients with HHCY in the ACS group compared to the non-CAD group (62.08% vs. 26.18%, *P* < 0.001). On the contrary, the HDL-C level and LVEF were significantly lower in ACS patients compared to non-CAD subjects.

**Table 1:** Baseline clinical characteristics of study participants

Characteristics	ACS (n = 828)	Non-CAD (n = 275)	*P*
Age (years)	33 (30–34)	32 (30–34)	0.206
Male, n (%)	795 (96.01)	245 (89.09)	**< 0.001**
SBP (mmHg)	126.04 ± 16.01	125.69 ± 14.66	0.64
DBP (mmHg)	77.71 ± 13.26	76.95 ± 11.48	0.297
HR (bpm)	75.73 ± 11.94	73.45 ± 12.09	**0.004**
Drinker, n (%)	148 (17.87)	56 (20.36)	0.361
*Medical history and coronary risk factors*			
Smoker, n (%)	572 (69.08)	139 (50.55)	**< 0.001**
BMI (kg/m^2^)	28.52 ± 4.64	26.80 ± 4.75	**< 0.001**
Hypertension, n (%)	402 (48.55)	92 (33.45)	**< 0.001**
Diabetes mellitus, n (%)	162 (19.57)	21 (7.64)	**< 0.001**
Hypertriglyceridemia, n (%)	468 (56.52)	89 (32.36)	**< 0.001**
Hypercholesterolemia, n (%)	257 (31.04)	52 (18.91)	**< 0.001**
High LDL-C, n (%)	237 (28.62)	44 (16.00)	**< 0.001**
Low HDL-C, n (%)	580 (70.05)	138 (50.18)	** < 0.001**
Family history of CAD, n (%)	116 (14.01)	24 (8.73)	**0.023**
Familial hypercholesterolemia, n (%)	25 (3.02)	1 (0.36)	**0.01**
Hyperuricemia, n (%)	367 (44.32)	97 (35.27)	**0.008**
Hyperhomocysteinemia, n (%)	514 (62.08)	72 (26.18)	**< 0.001**
Prior stroke, n (%)	4 (0.48)	0 (0.00)	0.577
*Laboratory results*			
BUN (mmol/L)	4.94 ± 1.39	4.79 ± 1.34	0.1
CR (µmol/L)	74.68 ± 15.14	74.69 ± 14.08	0.988
eGFR (mL/min/1.73 m^2^)	106.30 (92.80, 120.60)	103.40 (93.60, 116.20)	0.064
FBG (mmol/L)	5.35 (4.91–6.43)	5.04 (4.79–5.60)	**< 0.001**
HbA1c (%)	5.6 (5.2–6.2)	5.4 (5.1–5.7)	**< 0.001**
TG (mmol/L)	1.88 (1.31–2.89)	1.45 (1.01–2.18)	**< 0.001**
TC (mmol/L)	4.72 ± 1.78	4.34 ± 1.03	**< 0.001**
HDL-C (mmol/L)	0.91 ± 0.21	1.02 ± 0.22	**< 0.001**
LDL-C (mmol/L)	3.01 ± 1.61	2.65 ± 0.98	**< 0.001**
UA (µmol/L)	410.19 ± 96.67	385.83 ± 93.82	**< 0.001**
HCY (µmol/L)	16.55 (11.93–29.68)	12.50 (9.71–17.42)	**< 0.001**
hs CRP (mg/L)	3.11 (1.09–11.40)	1.12 (0.49–2.80)	**< 0.001**
*Cardiac function*			
LVEF	60 (54–65)	66 (62–68)	**< 0.001**

Data are expressed as mean ± standard deviation, medians with interquartile range or number (%)

*CAD* coronary artery disease, *ACS* acute coronary syndrome, *SBP* systolic blood pressure, *DBP* diastolic blood pressure, *HR* heart rate, *BMI* body mass index, *BUN* blood urea nitrogen, *CR* creatinine, *eGFR* estimated glomerular filtration rate, *FBG* fasting blood glucose, *HbA1c* glycosylated hemoglobin, *TG* triglyceride, *TC* total cholesterol, *HDL-C* high-density lipoprotein cholesterol, *LDL-C* low-density lipoprotein cholesterol, *UA* uric acid, *HCY* homocysteine, *hs CRP* high-sensitivity C-reactive protein, *LVEF* left ventricular ejection fraction

Bold values indicate statistical significance

### Univariate analysis of different ACS risk factors

As shown in Table 2, traditional ACS risk factors such as male gender, BMI, current smoker status, hypertension, DM, hypertriglyceridemia, hypercholesterolemia, High LDL-C, low HDL-C, family history of CAD, and familial hypercholesterolemia were obviously associated with the occurrence of ACS (*P* < 0.05). On the contrary, age, eGFR and drinker status showed no significant association with ACS. Moreover, non-traditional risk factors, such as hyperuricemia, especially HHCY, were also apparently related to ACS (for HHCY, OR, 4.615; 95% CI, 3.408–6.250; *P* < 0.001).

**Table 2:** Univariate logistic regression analysis of the association of ACS with variables

Variables	OR	95% Cl	*P*
Age	0.970	0.925–1.017	0.206
Male	3.628	2.199–5.985	**< 0.001**
BMI	1.104	1.067–1.142	**< 0.001**
eGFR	1.005	0.999–1.012	0.101
Drinker	0.851	0.604–1.200	0.357
Smoker	2.186	1.655–2.188	**< 0.001**
Hypertension	1.712	1.287–2.277	**< 0.001**
Diabetes mellitus	2.875	1.783–4.634	**< 0.001**
Hypertriglyceridemia	2.717	2.038–3.622	**< 0.001**
Hypercholesterolemia	1.930	1.380–2.700	**< 0.001**
High LDL-C	2.105	1.475–3.005	**< 0.001**
Low HDL-C	2.322	1.756–3.070	**< 0.001**
Family history of CAD	1.704	1.073–2.706	**0.024**
Familial hypercholesterolemia	8.531	1.15–63.252	**0.036**
Hyperuricemia	1.461	1.101–1.938	**0.009**
Hyperhomocysteinemia	4.615	3.408–6.250	**< 0.001**

*CAD* coronary artery disease, *ACS* acute coronary syndrome, *BMI* body mass index, *eGFR* estimated glomerular filtration rate, *HDL-C* high-density lipoprotein cholesterol, *LDL-C* low-density lipoprotein cholesterol, *OR* odds ratio, *CI* confidence interval

Bold values indicate statistical significance

### Multivariate logistic regression analysis of different ACS risk factors

Multivariate analysis with stepwise method further indicated that variables including male gender, BMI, eGFR, current smoker status, DM, hypertriglyceridemia, high LDL-C, and low HDL-C were identified as the independent predictors associated with the occurrence of ACS among young adults, while hypercholesterolemia, hypertension, family history of CAD, familial hypercholesterolemia, and hyperuricemia were not. After adjusting for the traditional risk factors mentioned above, HHCY was also significantly related to the presence of ACS in young subjects (OR, 4.561; 95% CI, 3.288–6.327; *P* < 0.001) (Table 3).

**Table 3:** Multivariate logistic regression analysis of different ACS risk factors

Variables	OR	95% Cl	*P*
Male	1.930	1.021–3.649	**0.043**
BMI	1.069	1.030–1.109	**< 0.001**
eGFR	1.014	1.006–1.022	**0.001**
Smoker	1.563	1.124–2.174	**0.008**
Diabetes mellitus	2.594	1.539–4.372	**< 0.001**
Hypertriglyceridemia	1.910	1.383–2.636	**< 0.001**
High LDL-C	1.728	1.166–2.562	**0.006**
Low HDL-C	1.926	1.399–2.651	** < 0.001**
Hyperhomocysteinemia	4.561	3.288–6.327	** < 0.001**

*ACS* acute coronary syndrome, *BMI* body mass index, *eGFR* estimated glomerular filtration rate, *LDL-C* low-density lipoprotein cholesterol, *HDL-C* high-density lipoprotein cholesterol, *OR* odds ratio, *CI* confidence interval

Bold values indicate statistical significance

### Clinical characteristics of young ACS patients in normal homocysteine and hyperhomocysteinemia groups

Young ACS patients were divided into two groups based on HCY levels (≤ 15, > 15 µmol/L). As shown in Table 4, young patients with HHCY were more likely to be male (97.28% vs. 93.95%, *P* = 0.026). In the HHCY group, the levels of CR and UA were elevated, while the level of eGFR was decreased and the prevalence of DM was lower. Moreover, the HHCY group had an increased prevalence of ST-segment elevation myocardial infarction (STEMI) (*P* = 0.041), multi-vessel disease (*P* = 0.036), and decreased value of LVEF (*P* = 0.01). In addition, the Gensini Score was also obviously elevated in the HHCY group (*P* = 0.043).

**Table 4:** Clinical characteristics of young ACS patients according to homocysteine levels

Characteristics	HCY ≤ 15 µmol/L (n = 314)	HCY > 15 µmol/L (n = 514)	*P*
*Baseline characteristics*			
Age (years)	33 (30–34)	32 (30–34)	0.303
Male, n (%)	295 (93.95)	500 (97.28)	**0.026**
Drinker, n (%)	64 (20.38)	84 (16.34)	0.161
*Traditional coronary risk factors*			
BMI (kg/m^2^)	28.38 ± 4.09	28.60 ± 4.95	0.553
Smoker, n (%)	215 (68.47)	357 (69.46)	0.816
Hypertension, n (%)	154 (49.04)	248 (48.25)	0.830
Diabetes mellitus, n (%)	91 (28.98)	71 (13.81)	** < 0.001**
Family history of CAD, n (%)	41 (13.06)	75 (14.59)	0.606
*Laboratory results*			
TG (mmol/L)	1.84 (1.26–3.06)	1.94 (1.36–2.84)	0.571
TC (mmol/L)	4.80 ± 1.56	4.65 ± 1.69	0.213
HDL-C (mmol/L)	0.91 ± 0.22	0.90 ± 0.20	0.729
LDL-C (mmol/L)	3.06 ± 1.55	2.97 ± 1.52	0.386
CR (µmol/L)	71.83 ± 13.30	76.16 ± 13.04	**< 0.001**
eGFR (ml/min/1.73 m^2^)	109.30 (96.80–126.00)	104.70 (91.10–118.65)	**0.002**
hs CRP (mg/L)	3.10 (1.12–11.9)	2.97 (0.98–11.77)	0.818
UA (µmol/L)	397.56 ± 89.78	417.65 ± 99.77	**0.004**
*Clinical type of ACS*			
STEMI, n (%)	100 (31.85)	201 (39.11)	**0.041**
NSTEMI, n (%)	73 (23.25)	94 (18.29)	0.090
UAP, n (%)	141 (44.90)	219 (42.61)	0.563
*CAG characters*			
None	25 (7.96)	24 (4.67)	0.068
Single-vessel, n (%)	142 (45.22)	209 (40.66)	0.218
Double-vessel, n (%)	71 (22.61)	132 (25.68)	0.360
Triple-vessel, n (%)	76 (24.20)	149 (28.99)	0.147
Multi-vessel, n (%)	147 (46.82)	281 (54.67)	**0.036**
Gensini Score	30 (12–48)	32 (16–62)	**0.043**
*Cardiac function*			
LVEF	60 (56–65)	60 (52–65)	**0.01**

Data are expressed as mean ± standard deviation, medians with interquartile range or number (%)

*CAD* coronary artery disease, *ACS* acute coronary syndrome, *BMI* body mass index, *HCY* homocysteine, *TG* triglyceride, *TC* total cholesterol, *HDL-C* high-density lipoprotein cholesterol, *LDL-C* low-density lipoprotein cholesterol, *CR* creatinine, eGFR estimated glomerular filtration rate, *UA* uric acid, *STEMI* ST-segment elevation myocardial infarction, *NSTEMI* non ST-segment elevation myocardial infarction, *UAP* Unstable Angina Pectoris, *CAG* coronary angiography, *hs CRP* high-sensitivity C-reactive protein, *UA* uric acid, *LVEF* left ventricular ejection fraction

Bold values indicate statistical significance

### Serum HCY level in different groups of young ACS patients

**Fig. 2:**
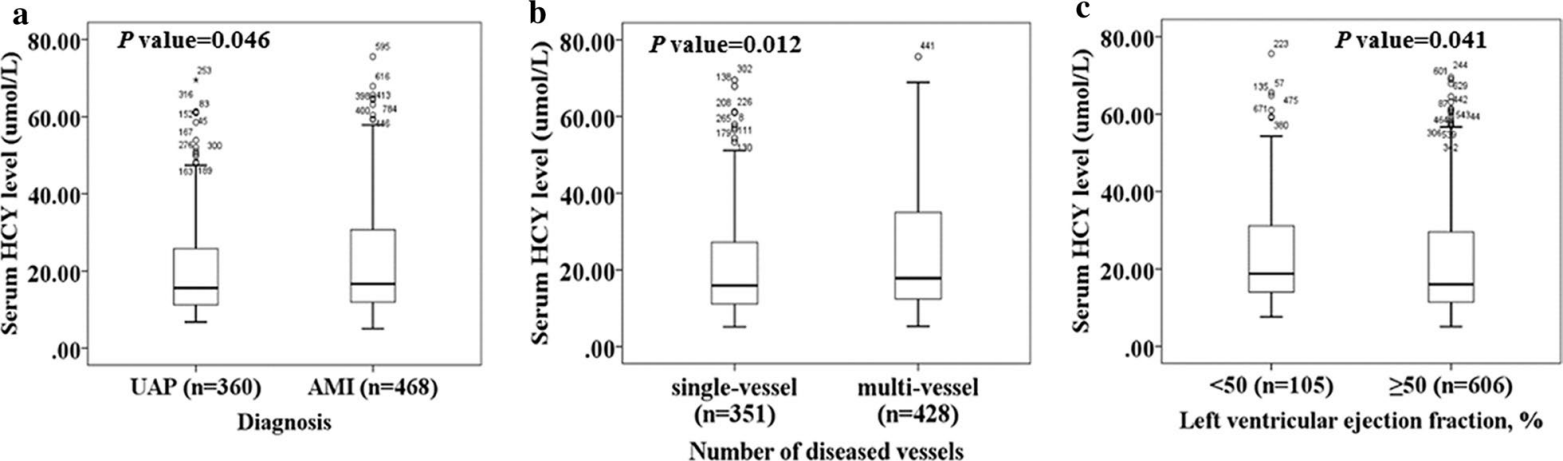
Comparison of the serum HCY level in different groups among ACS patients. **a** Comparison of the serum HCY level between patients with UAP and AMI. **b** Comparison of the serum HCY level between patients with single-vessel and multi-vessel disease. **c** Comparison of the serum HCY between patients with LVEF ≥ 50% and LVEF < 50%. *HCY* homocysteine, *UAP* unstable angina pectoris, *AMI* acute myocardial infarction, *ACS* acute coronary syndrome, *LVEF* left ventricular ejection fraction

As shown in Fig. 2a, the serum HCY was higher in patients with AMI (*P* = 0.046). Moreover, 49 ACS patients had no lesion coronary arteries (major coronary arteries with luminal diameter stenosis ≥ 50%) according to coronary angiography. So, except for these patients, the others were classified into a single-vessel group (n = 351) and multi-vessel group (n = 428). Similarly, as shown in Fig. 2b, increased HCY level was observed in the multi-vessel group (*P* = 0.012). Finally, since LVEF data were lost for 117 patients, ACS patients were divided into LVEF ≥ 50% group (n = 606) and LVEF < 50% group (n = 105). Figure 2c indicated that serum HCY was elevated in LVEF < 50% group.

### Correlation of serum HCY levels with Gensini Scores in young ACS patients

**Fig. 3:**
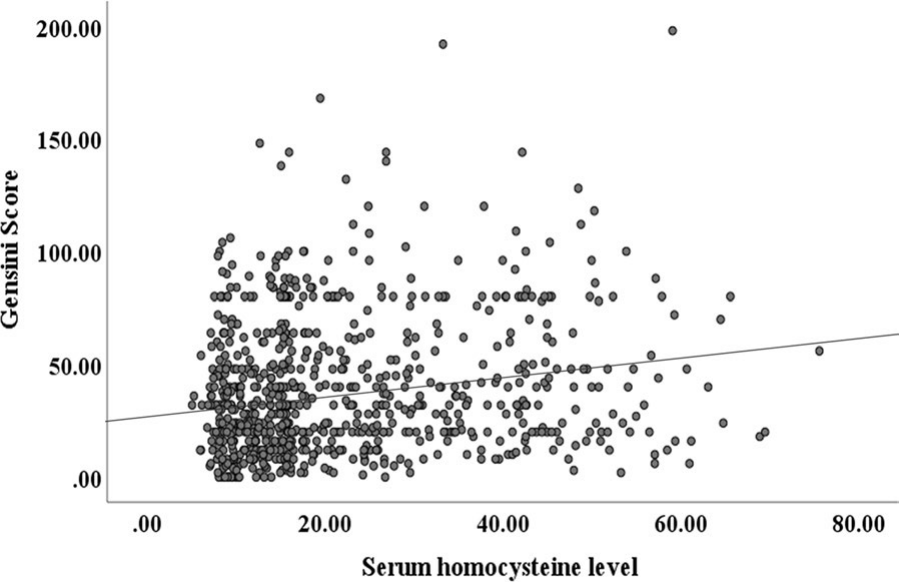
Correlation of serum homocysteine levels with respective Gensini Scores (r = 0.142, *P* < 0.001)

Figure 3 showed that serum HCY levels were significantly correlated with Gensini Score in ACS patients (r = 0.142, *P* < 0.001).

## Discussion

In this observational study that included young patients who were ≤ 35 years of age, HHCY was found to be significantly associated with the presence of ACS, which was independent of traditional risk factors. In addition, HHCY had a strong correlation with the severity of coronary artery stenosis.

Due to lifestyle changes, especially the increased obesity and reduced physical activity, the onset age for CAD has been gradually decreasing [17]. A previous study showed that nearly 4–10% of AMI patients were younger than 45 years old [18]. In contrast, the prevalence of AMI among young patients (< 35 years old) in China has doubled over a decade [19], causing serious consequences for families and society. Compared with older patients, younger ones may have different coronary risk factor profiles. Results from a review identified male gender, current smoking status, alcohol consumption, diabetes, hypertension, dyslipidemia, psychosocial factors, sedentary lifestyle, obesity, and family history of premature MI as the leading causes of ACS in most young patients [20]. In the current study, the age range of young participants were narrowed to 18–35 years to determine the association between HHCY and ACS. The results showed that young ACS patients were more likely to have HHCY.

Studies performed over the last two decades identified HHCY as a crucial promoter for atherosclerotic vascular disease. There is a great controversy on the association between HHCY and the incidence of CAD, and whether it is casual, since lowering HCY levels in patients with CAD has not shown any benefit [4]. Nevertheless, many observational studies found that HHCY, which acted as an important marker, was strongly associated with CAD and major adverse cardiac events (MACE) (death, reinfarction, restenosis) after PCI [5]; however, most of these studies were conducted in older people. Despite the increasing population of young ACS and the growing proportion of sudden death among these patients, there is limited evidence on the effect of HHCY on the risk of ACS in young adults. Additionally, the results of a few available studies were conflicting. A case–control study carried out among patients aged < 40 years showed a positive correlation between HHCY and CAD occurrence [21]. In comparison, another study showed no difference of serum HCY level between healthy controls and young AMI patients aged ≤ 35 years [22]. Thus, this large-scale observational study was conducted, which revealed that young patients with ACS had higher HCY level than non-CAD participants [16.55 (11.93–29.68) vs. 12.50 (9.71–17.42), *P* < 0.001], and HHCY was identified as an independent predictor associated with the presence of ACS (OR, 4.561; 95% CI, 3.288–6.327; *P* < 0.001).

Except for HHCY and other conventional risk factors, the present study found eGFR was also associated with ACS among young adults (OR, 1.014; 95% CI, 1.006–1.022; *P* < 0.001). This result was consistent with the study of Sasso et al. [23], which suggested a liner correlation between eGFR and cardiovascular events. But Sasso et al. study was conducted in DM patients who were much older. In addition, eGFR was decreased in patients with HHCY in the study, while the prevalence of DM was identified to be lower, which was unexpected. A study involving 3056 middle-aged and elderly patients with CAD or Heart valve disease suggested there was no correlation between DM and plasma HCY concentrations [24]. Glowinska et al. [25] investigated new atherosclerosis risk factors in diabetic children and adolescents, and found diabetic patients had lower concentrations of HCY compared with healthy subjects, but with no statistical significance. Giannattasio and colleagues [26] showed young adults with type 1 diabetes mellitus without microvascular complications had significant lower HCY levels and obvious higher vitamin B12 concentrations compared with controls. Furthermore, previous study indicated plasma HCY levels in young individuals at increased risk of type 2 diabetes mellitus were associated with subtle differences in GFR, but not with insulin resistance [27]. In this study, due to the young age and exclusion of lower eGFR in the participants, eGFR of most patients were in a normal range, which may partially affect the correlation between HHCY and DM. Since plasma HCY levels are determined by genetic and nutritional factors, the association of HHCY and DM in young ACS patients need more in-depth research.

The relationship between HCY and the severity of coronary artery stenosis has been investigated by several studies before. Still, the current study is the only one conducted among the young ACS population. The results of this study showed a positive correlation between HHCY and angiographic severity expressed by Gensini Score. Li et al. [28] studied 667 middle-aged and elderly CAD patients who underwent drug-eluting stent implantation and reported that patients with HHCY had a higher stenosis degree, as indicated by elevated SYNTAX scores. In their study, Shenoy et al. [29] suggested that serum HCY level was significantly correlated with the Gensini Score of CAD patients (r = 0.443), which was consistent with this study. However, the sample size in the study conducted by Shenoy et al. [29] was smaller, and the participants of Li et al. [28] and Shenoy et al. [29] study were much older. In addition, Li et al. [28] also showed that the number of coronary artery target vessels in the HHCY group was obviously higher, and patients with high HCY levels had a higher proportion of coronary lesions. Another study involving HCY levels and premature CAD (56.1 ± 6.2 years of age) in 2019 [30] showed that the HCY levels were significantly higher in patients with multi-vessel disease. These findings were consistent with the current study on the association between HHCY and the number of lesion vessels. Nonetheless, their participants were older than the participants in the present study. In this study, decreased value of LVEF was found in young ACS patients with HHCY, which might be due to the relatively high prevalence of AMI in patients with higher HCY.

Many possible mechanisms have been reported as relevant for the association between HCY and CAD. A recent review [31] showed that HCY had a vast array of toxic effects on the vasculature, including impairing endothelial function by reducing the production of nitric oxide (NO), inducing vascular remodeling and vessel stiffening by increasing the synthesis of smooth muscle cells (SMC), as well as elevating adventitial inflammation, which might lead to the development of atherosclerosis. This review [31] also hypothesized that besides serum HCY, tissue-bound HCY and the incorporation of HCY into proteins could cause toxicity to the vasculature. In their study, Yun et al. [32] indicated the enhancement of arterial stiffness in HHCY might be attributed to HCY-related LDL atherogenesis, such as small LDL particle size and its oxidative modification. Bianca et al. [33] suggested that HCY exerted a prothrombotic effect by enhancing platelet aggregation. Several studies also showed that HHCY might enhance the adverse effects of CAD risk factors such as essential hypertension, smoking, dyslipidemia, and diabetes mellitus [34–37]. These were probably related to the formation and progression of CAD in young adults. Furthermore, some pathophysiological mechanisms of acute coronary damage are often not taken into consideration which, although relevant, we do not yet know adequately [38, 39].

Although traditional risk factors have a vital role developing cardiovascular disease, only 50% of these diseases could be explained by classical factors, which is why non-traditional risk factors have drawn more attention. The clinical significance of this article is to identify that HHCY plays an important role in the occurrence and progression of ACS, which increased awareness of the importance of the HCY level among patients ≤ 35 years of age. Since excessive weight, current smoker status, alcohol and caffeine intake, and insufficient vitamin B and folic acid levels could increase HCY concentration, young adults should adhere to a healthy lifestyle so as to maintain HCY levels within the normal range.

### Limitations

Our study has a few limitations. First, this was a retrospective study. Although the serum HCY levels are mainly determined by vitamin B and folic acid intake, vitamin B and folate levels were not measured in the study. Second, albuminuria has been identified to correlate linearly with cardiovascular risk, especially combined with eGFR [23, 40]. Due to the retrospective nature of this study, albuminuria was not dosed and unable to be analyzed in the study. Third, since all the medical history data of participants were obtained from electronic medical records, it was hard to ensure that they were accurate. Finally, the majority of patients with ACS who had high homocysteine levels were males; therefore, the results had limited value for the young female population.

## Conclusion

HHCY is significantly associated with the presence of ACS and the severity of coronary artery stenosis in young adults ≤ 35 years of age.

## Data Availability

The datasets used and/or analyzed during the current study are available from the corresponding author on reasonable request.

## Abbreviations

ACS: Acute coronary syndrome
HCY: Homocysteine
CAD: Coronary artery disease
CAG: Coronary angiography
HHCY: Hyperhomocysteinemia
STEMI: ST-segment elevation myocardial infarction
LVEF: Left ventricular ejection fraction
BMI: Body mass index
AMI: Acute myocardial infarction
FRFs: Framingham risk factors
TG: Triglycerides
TC: Total cholesterol
HDL-C: High-density lipoprotein cholesterol
UA: Uric acid
SBP: Systolic pressure
FH: Familial hypercholesterolemia
LVEF: Left ventricular ejection fraction
SD: Standard deviation
IQR: Interquartile range
MACE: Major adverse cardiac events
